# Comparison of SNR efficiencies and strain for cine DENSE using conventional EPI, flyback EPI and spiral k-space trajectories

**DOI:** 10.1186/1532-429X-13-S1-P58

**Published:** 2011-02-02

**Authors:** Xiaodong Zhong, Bruce S Spottiswoode, Craig H Meyer, Frederick H Epstein

**Affiliations:** 1 Siemens Healthcare, Lilburn, GA, USA; 2 MRC/UCT Medical Imaging Research Unit, University of Cape Town, Cape Town, South Africa; 3 Biomedical Engineering and Radiology, University of Virginia, Charlottesville, VA, USA

## Introduction

The original implementation of 2D cine DENSE (displacement encoding with stimulated echoes) employed a conventional EPI *k*-space trajectory for rapid data sampling. Follow-up studies used flyback EPI to reduce image artifacts. More recently a spiral *k*-space trajectory was utilized for improved SNR.

## Purpose

To evaluate and compare SNR efficiencies and strain results of these three techniques for 2D cine DENSE imaging.

## Methods

Cine DENSE pulse sequences were developed that employed different *k-*space trajectories, namely conventional bottom-up interleaved EPI, flyback bottom-up interleaved EPI, and interleaved spiral. They were compared in volunteers using a two-breath-hold protocol on a 1.5T Siemens Avanto system with a four-channel chest coil. In accordance with protocols approved by the local institutional review board, 5 healthy volunteers were imaged. Identical parameters included pixel size = 3.8 × 3.8 mm^2^, slice thickness = 8 mm, flip angle = 20°, cardiac phases = 15, displacement encoding frequency = 0.08 cycles/mm, two-point phase cycling, and through-plane dephasing frequency = 0.08 cycles/mm to suppress artifacts. Other parameters for conventional/flyback EPI included image matrix = 72 × 96, TE = 8.57/10.57 ms, TR = 17.69/21.19 ms, ETL = 8, segments = 16, sampling time per image = 99.8 ms, heartbeats per breath-hold = 21/20, and fat suppression by water excitation. For spiral, other parameters included image matrix = 96 × 96, TE = 1.08 ms, TR = 17 ms, interleaves = 6, interleaves per heartbeat = 2, sampling time per image = 66.8 ms, heartbeats per breath-hold = 14, and fat suppression by chemically-selective saturation pulses applied prior to displacement-encoding pulses. Based on the magnitude-reconstructed images, SNR efficiencies were calculated. Strain maps were also calculated.

## Results

**Figure 1:**
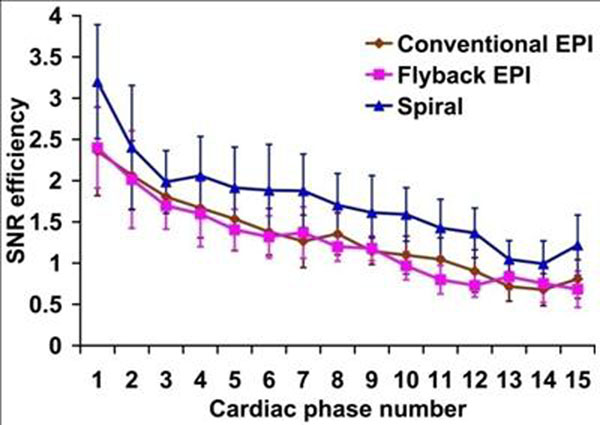
Mean SNR efficiency curves of three techniques.

As shown in Figure 1, there is a small difference in SNR efficiencies between conventional and flyback EPI, which is due to their small TE and TR differences. In contrast, the SNR efficiency of spiral increases by about 33% at early phases and about 79% at late phases compared to the EPI techniques, which is attributed to shorter TE and more efficient sampling. The circumferential strain (Ecc) was calculated for mid-ventricular segments at end-systole (Table 1). One-way ANOVA analysis showed no statistically significant difference among three techniques.

**Table 1:** The end-systolic Ecc of mid-ventricular segments measured with three techniques.

	Mid anterior	Mid lateral	Mid posterior	Mid inferior	Mid septum	Mid anterior septum
**Conventional EPI DENSE**	-0.20 ± 0.02	-0.22 ± 0.04	-0.19 ± 0.05	-0.18 ± 0.02	-0.17 ± 0.01	-0.17 ± 0.02
**Flyback EPI DENSE**	-0.19 ± 0.03	-0.22 ± 0.05	-0.21 ± 0.03	-0.18 ± 0.02	-0.14 ± 0.01	-0.15 ± 0.02
**Spiral DENSE**	-0.19 ± 0.03	-0.21 ± 0.03	-0.21 ± 0.02	-0.17 ± 0.02	-0.15 ± 0.03	-0.15 ± 0.03

## Conclusions

Conventional EPI, flyback EPI and spiral cine DENSE produce similar strain results. Spiral cine DENSE provides improved SNR efficiency compared to the other two techniques.

